# The effect of aqueous and ethanolic extract of Iranian propolis on Candida Albicans isolated from the mouth of patients with colorectal malignancy undergone chemotherapy: An in-vitro study

**DOI:** 10.22088/cjim.11.1.62

**Published:** 2020

**Authors:** Fatemeh Sayyadi, Saeed Mahdavi, Ali Akbar Moghadamnia, Dariush Moslemi, Atena Shirzad, Mina Motallebnejad

**Affiliations:** 1 Department of Oral Medicine, Dental School, Babol University of Medical sciences, Babol, Iran; 2 Infectious Diseases and Tropical Medicine Research Center, Health Research Institute, Babol University of Medical sciences, Babol, Iran; 3 Department of Medical Parasitology and mycology Babol university of Medical Sciences, Babol, Iran; 4 Neuroscience Research Center, Health Research Institute, Babol University of Medical Sciences, Babol, Iran; 5 Cancer Research Center, Health Research Institute, Babol University of Medical Sciences, Babol, Iran; 6 Oral Health Research Center, Health Research Institute, Babol University of Medical Sciences, Babol, Iran

**Keywords:** Candida albicans, propolis, chemotherapy, 5-fu, antifungal

## Abstract

**Background:**

Candidiasis is one of the most common fungal infections in immunosuppressed patients. The condition is usually treated with local and systemic antifungal agents. Given the antifungal properties of propolis, it appears this natural resin material can be effective in treating this infection. The aim of the present in vitro study was to compare the effect of Iranian propolis with those of routine antifungal agents on Candida species isolated from the oral candida lesions of patients with cancer, who had undergone chemotherapy, and a standard strain of Candida albicans.

**Methods:**

A total of 23 samples were collected from the oral cavities of patients with colorectal cancer, who had undergone chemotherapy with 5-fu. The fungal species were determined based on the results of culture in C. albicans chromagar medium, formation of the germ tube and formation of vesicles. The MIC of aqueous extract propolis (AEP) and ethanolic extract of propolis (EEP) and amphotericin B (AMP-B), fluconazole (FL) and nystatin (NYS) were compared.

**Results:**

A total of 23 oral C. albicans samples were isolated. The MICs of FL and AMP- B were similar and less than those of EEP, AEP and NYS (P<0.001). In addition, the MIC of AEP was higher than EEP (P<0.001). The MIC of AMP- B on the strains isolated from the patients was more than that of the standard strain (P=0.012).

**Conclusion:**

The aqueous and ethanolic extracts of Iranian propolis exhibited antifungal activity, with a greater effect of the EEP compared to the AEP.

Candidiasis is the most common oral fungal infections (1). There are many predisposing factors, including, chemotherapy, immunosuppressive conditions, mucosal injuries and deceased salivary flow (2). In patients with cancer the most common types of candidiasis are pseudomembranous and erythematous (3). A systematic review showed prevalence of clinical fungal infections during chemotherapy is 38% (4), which is attributed to damage of the mucosal barrier and granulocytopenia due to chemotherapy and long-term use of broad-spectrum antibiotics and corticosteroids (5,6). On the other hand, Sepulveda et al reported30% of oral lesions in patients undergoing chemotherapy had clearly been produced by C. albicans (5). To treat oral candidiasis, local antifungal agents, such as NYS mouthwashes and clotrimazole and FL lozenges are used; however, these agents have various effects (7, 8). Injudicious use of antifungal agents can result in drug resistance (9). 

Systemic medications are used to treat refractory fungal infections in immunosuppressed patients (4). However, these medications have many side effects and may cause drug interactions (10). Propolis or honeybee wax is a resin material that is collected by honeybees from different plant sources (11). Propolis mainly consists of resin, gum, phenol aldehydes (polyphenols), wax and essential fatty acids. Phenolic acid, esters and flavonoids are the most important constituents of propolis and various biologic properties of propolis, including its anti-inflammatory, antifungal, antibacterial and antiviral activities, are attributed to these constituents. These properties have made propolis as a choice for therapeutic purposes (12). 

The antifungal activity of propolis against various Candida species has been evaluated in many studies (10, 12). The results have shown that the EEP can be an alternative for the treatment of candidiasis in HIV patients (13). In addition, it has been demonstrated that propolis can be an alternative treatment modality for recurrent candidiasis, especially in the elderly and in immunosuppressed patients (14). 

There are many reports on increasing Candida species resistant to antifungal agents in patients undergoing chemotherapy (1). As oral candidiasis is prevalent in patients treated with 5-fu (15). The present in vitro study was designed to compare the antifungal effects of Iranian propolis and other antifungal agents such as NYS, AMP-B and FL on Candida species isolated from oral candidiasis in patients with 5-fu chemotherapy*.*

## Methods

In the present in vitro study, 23 patients with oral candidiasis were evaluated. The samples were collected from the oral candida lesions of patients who had referred to Shahid Rajaee Hospital in Babolsar for chemotherapy from November 2014 to March 2015. All the patients (30-65 years old) had colorectal cancer, and a chemotherapy regimen of folfox (5-Fu, leucovein eloxatin). Exclusion criteria consisted of systemic diseases such as diabetes, active oral bacterial infections, vascular collagen diseases, smoking, stage 4 cancer, a history of radiotherapy and chemotherapy, use of antibiotics and antifungal agents during the previous two weeks and a history of corticosteroid use before chemotherapy. 

Propolis was prepared in two forms of 25% AEP and 50% EEP from Suren Tak Tous Company; NYS (APP Lichem, Germany), AMP-B (Sigma, USA) and FL (Sigma, USA) were provided from Suren Pharmaceutical Company. The standard strain of C. albicans (ATCC) was used to compare.

**Study procedure: **This project was approved by the Ethics Committee no. 5262 of VP of Research Babol University of Medical Sciences. Informed consent was taken from all patients.

Candida's samples were collected from the lesions on the tongue, palate and buccal mucosa of the patients with the use of a sterile swab impregnated with sterile saline solution. Then the samples were cultured on plates containing Sabourad’s dextrose agar (Himedi, India) with chloramphenicol (Sc) using the linear technique. The samples were transferred to the Mycology and Parasitology Laboratory of Babol University of Medical Sciences under sterile transfer conditions. To identify C. albicans, we used the methods of Chromogenic Candida Agar (CCA), vesicle formation and germ tube technique (16, 17, 18).

All the colonies received from the patients and the standard strain of C. albicans were subcultured in Sc medium and after growth, a suspension of yeast cells was prepared with the use of 2 mL of sterile physiologic serum (normal saline) in a shaker. Then McFarland’s 0.5 standard was used to reach a concentration of 1×10^6^-5×10^6^ cells per Ml (19). Then RPMI 1640 medium was used to reach working dilutions of 5×10^2^-2.5×10^3^.

To determine the minimum inhibitory concentration (MIC), the microdilution technique recommended by Clinical and Laboratory Standards Institute (CLSI-M27-A3) for the RPMI 1640 medium and 96-well sterile microplates were used (20). To this step NYS, AMP-B, FL, AEP and EEP were prepared in 9 dilutions. NYS, AMP-B and FL were prepared at concentrations 0.25-128, 0.310-16 and 0.125-64µg/ml, respectively; and AEP and EEP were prepared at concentrations of 0.4-210 and 0.2-130mg/ml, respectively. 

 Dimethyl sulfoxide (DMSO) control solvent was used at a maximum concentration of 2 µL in each well (20). The negative control wells without any fungal growth (200 mL of RPMI) and positive control wells containing fungi without any drug or extract were used to control growth. Ethanol, too, at a maximum concentration of 12.5%, was used as an EEP control and none exhibited any antifungal activity against the samples. All the procedures were carried out as duplicate. This test was repeated three times to minimize errors on the standard strain. All the microplates were incubated at 35°C for 48 hours and finally the microplates were inspected visually for turbidity or translucency. Turbidity indicated the presence of fungi. Therefore, the last translucent well was considered MIC. 

Data were analyzed with SPSS Version 18. Normal distribution of variables was confirmed with the use of Shappiro-Wilk test. ANOVA and posthoc Tukey tests were used to compare data collected after application of different treatment modalities. Non-paired t-test was used to compare MIC of each fungal strain in each group with the standard strain. Statistical significance was set at P<0.05. 

## Results

In the present study, Candida samples were cultured from 23 patients with colorectal cancer, who had clinical oral candidiasis. C. albicans was isolated from 22 samples and the other one was C. glabrata. The Candida species were differentiated based on the formation of germ tube, formation of vesicles and the results of culturing in the C. albicans chromagar culture medium. 

**Figure 1:**
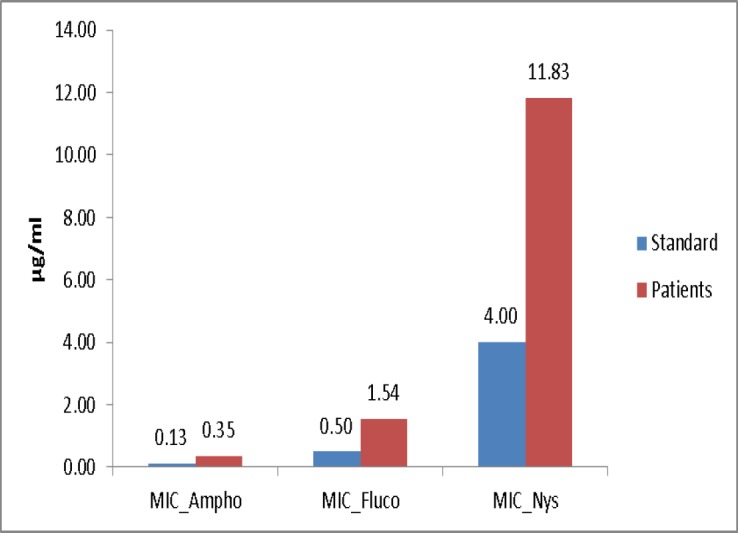
MICs of antifungal agents in samples collected from the patients and standard strain

**Figure 2:**
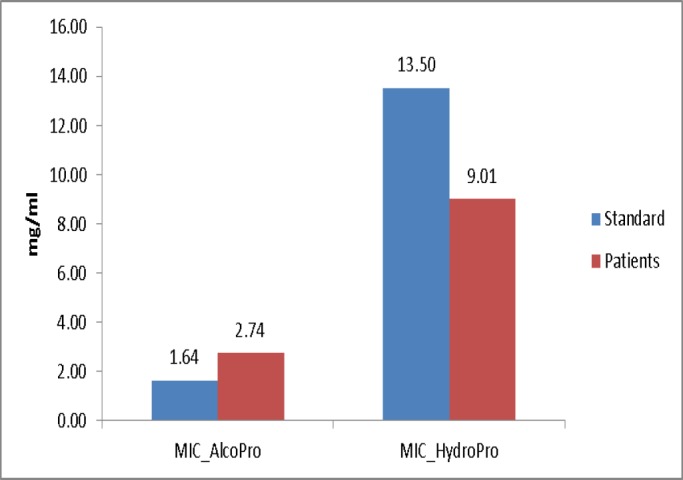
MICs of AEP and EEP in samples collected from the patients and standard strain

The results mainly achieved based on dilution of the liquid culture medium, showed that AMP-B, NYS, FL and AEP and EEP had inhibitory effects on C. albicans. The MICs for AMP-B, FL, NYS, EEP, and AEP were 0.35 µg/mL, 1.54 µg/mL, 11.83 µg/mL 2.74 mg/mL and 9.01 mg/mL, respectively. As shown in figures 1 and 2, the lower and higher concentrations belonged to AMP-B and AEP, respectively.

The results showed that the fungal inhibitory effect of AMP-B was similar to that of FL and its effect was higher than that of the NYS, EEP and AEP (P<0.001). In addition, the antifungal activity of EEP was higher than AEP (P<0.001).

Overall, both the EEP and AEP had inhibitory effects on fungi, with higher effect of EEP compared to the AEP (P<0.001). There were no significant difference in the MICs of FL, NYS and EEP and AEP between standard C. albicans and the candidiasis isolated from the patients, but higher doses of AMP-B were required to inhibit samples isolated from the patients’ oral cavities compared to the standard strain (p=0.012).

## Discussion

In the present study, the antifungal effects of the AEP and EEP were compared with those of routine antifungal medications AMP-B, FL and NYS. Totally, the minimum inhibitory concentrations (MICs) of AMP-B and FL were similar to each other but less than those of NYS, EEP and AEP, moreover, the MIC of EEP was lower than that of AEP. The compositions of different propolis products are different depending on plant species, local climate and environment, resulting in differences in the biologic properties of propolis in different geographic locations; however, the antifungal activity of this material has been show (21) and since propolis is a natural agent, its antifungal effects can be used with higher dose for patients. The antifungal properties of propolis are mainly attributed to its flavonoid (polyphenol) and cinnamic acid contents (22). It has been shown that propolis inhibits DNA replication in fungi, and indirectly inhibits cellular division (10). On the other hand, Wander et al. in 2008 showed that propolis can reduce the Candida adhesion on denture surface more than fluconazole and nystatin (23).

 Thus, it might be used as an ideal combination for the treatment of fungal infections (24). A synergistic effect was observed for the action of EEP in combination with ﬂuconazole and voriconazole against C. albicans by Katarzyna in 2018 (25). Christian evaluated MICs of six different commercial extracts of propolis and showed that despite differences in polyphenol concentrations, all of them were able to prevent the growth of C. albicans (22). Martin indicated EEP had effect on C. albicans isolated from patients with AIDS (13). An in vitro study in 2016 has shown that propolis has signiﬁcant antifungal activity, which is comparable with ﬂuconazole and*** i****traconazole* against yeasts isolated from blood culture in adult patients in ICUs (26). In the present study, the antifungal effect of propolis on samples isolated from immunosuppressed individuals was also evaluated. It was shown that both the EEP and AEP have antifungal activity on the standard strain, and C. albicans strains isolated from the mucosa of individuals who were immunosuppressed. Since in previous studies the AEP has been used less frequently, in this study two different extracts were used and compared. The EEP exhibited greater antifungal activity compared to the AEP, which might be attributed to different ability of solvents (water or alcohol) to extract flavonoid components from propolis. Ethyl alcohol can provide more flavonoids than water (27). 

In a study in 2006 by Mello, the MIC of 20% EEP against C. albicans was similar to that of NYS (18). However, in the present study, EEP exhibited lower antifungal activity compared to NYS. It appears such difference is due to differences in geographical locations, plant species and climates in the origins of propolis in this study. The higher percentage of alcohol in this propolis extract. Since the bulk of propolis consists of resin and wax, a higher concentration of alcohol can result in greater release of these soluble substances (12).

In the present study, the MIC of AMP-B on C. albicans strains isolated from the oral candida lesions was higher than the standard strain of C. albicans, which means higher concentration of AMP-B was needed to inhibit C. albicans strain isolated form patients. According to the MIC of AMP-B and EEP on standard and isolated strains of C. albicans, higher doses of AMP-B are needed to inhibit C. albicans strains isolated from immunosuppressed patients than standard strain of which this increase in dose is not needed for EEP.

**In conclusion t**he aqueous and ethanolic extracts of Iranian propolis exhibited antifungal activity but the ethanolic extract was more effective than the aqueous extract. In addition, the EEP, compared to AMP-B exhibited antifungal activity against both C. albicans species isolated from the patients and the standard C. albicans species. 
